# Reliability of Automated Cephalometric Analysis: A Comparative Assessment of Stratification Strategies Based on Chronological Age Versus Dentition Stage

**DOI:** 10.3390/dj14030167

**Published:** 2026-03-12

**Authors:** Anh Thi Ngoc Do, Hung Trong Hoang, Hieu Ngoc Le, Thuy-Trang Thi Ho

**Affiliations:** 1 Faculty of Dentistry, University of Medicine and Pharmacy at HCM City, Ho Chi Minh City 700000, Vietnam; dtnanh@ntt.edu.vn (A.T.N.D.); htrhung@ump.edu.vn (H.T.H.); 2 Faculty of Dentistry, Nguyen Tat Thanh University, Ho Chi Minh City 700000, Vietnam; 3 Faculty of Information Technology, Van Hien University, Ho Chi Minh City 700000, Vietnam; hieuln@vhu.edu.vn

**Keywords:** cephalometry, artificial intelligence, automatic cephalometric analysis, WebCeph, manual tracing, dentition stage, chronological age, mixed dentition, reliability

## Abstract

**Objectives**: This study evaluated the accuracy of an artificial intelligence (AI)-based cephalometric software (WebCeph version 2.0.0.) compared with manual tracing and determined whether stratifying patients by chronological age or dentition stage provides a more clinically relevant assessment of AI accuracy. **Methods**: Three hundred lateral cephalometric radiographs of Vietnamese patients were traced manually by an orthodontist (reference standard) and analyzed automatically by WebCeph. Intra-observer reliability was validated using ICC and Dahlberg’s error. We analyzed the data using three stratification strategies: (1) Overall; (2) Chronological age (<18, 18–25, >25 years); and (3) Dentition stage (<9 primary-early mixed, 9–12 late mixed, >12 permanent). The primary outcome was the absolute measurement difference (∣*Δ*∣), analyzed using the Kruskal–Wallis test and effect size ($\eta^{2}$). **Results**: Overall, WebCeph showed high concordance with manual tracing (*ICC* > 0.80 for most parameters). Chronological age stratification showed weak associations with measurement error; differences between groups were largely non-significant (p>0.05) with a small effect size ($\eta^{2}\approx 0.015$). In contrast, the dentition stage revealed significant performance disparities (p<0.05). Notably, accuracy for the Mandibular Arc (*ICC* = 0.349) and Mandibular Plane Angle (p=0.048) degraded significantly in the primary-early mixed group, a vulnerability obscured by chronological age-based stratification. **Conclusions**: Dentition stage is a more sensitive and biologically relevant predictor of AI accuracy than chronological age. While WebCeph is reliable for permanent dentition, accuracy degrades significantly in the primary-early mixed phase. Clinicians should prioritize manual verification of mandibular and incisor landmarks in mixed-dentition children.

## 1. Introduction

Cephalometric radiography remains a cornerstone of orthodontic diagnosis, treatment planning, and outcome assessment [1]. Traditionally, the manual tracing of these radiographs serves as the reference standard; however, this process is time-consuming and susceptible to inter- and intra-observer variability [2,3,4]. The advent of artificial intelligence (AI) has revolutionized this workflow, with automated landmark detection systems such as WebCeph (Assemble Circle, Gyeonggi-do, Republic of Korea) offering the potential to significantly reduce clinician workload and standardize measurements [5,6,7].

While the efficiency of AI-driven analysis is well-documented, the clinical adoption of these tools hinges on their rigorous validation against the manual standard. To date, numerous studies have reported generally high agreement between AI and human examiners [8,9]. However, two critical gaps remain in the current body of literature. First, most AI algorithms are trained on datasets predominantly composed of specific ethnic groups (e.g., Caucasian or East Asian populations). The performance of these models when applied to under-represented populations with distinct craniofacial morphologies, such as the Vietnamese [7,10]. Moreover, recent evidence suggests that scoping clinical accuracy is vital for global generalizability [11].

Second, and perhaps more importantly, there is a lack of consensus regarding the optimal stratification strategy for evaluating AI reliability in growing patients. Previous studies have typically assessed performance either on the overall sample or by stratifying patients based on chronological age [12,13]. While chronological age is a convenient demographic variable, it may not fully reflect the biological complexity of the craniofacial structures. The transition from mixed to permanent dentition introduces complex anatomical changes, including tooth germ superimposition and root resorption. These biological factors create ‘noise’ that may confound AI algorithms more significantly than chronological age [14,15,16]. It remains unclear whether stratifying patients by chronological age or by dentition stage provides a more clinically relevant predictor of AI accuracy.

Therefore, the aim of this study was to compare the accuracy of an AI-based cephalometric software (WebCeph) with manual tracing in a Vietnamese orthodontic cohort. Unlike previous investigations, this study specifically evaluates three distinct stratification strategies: (1) an overall analysis, (2) a chronological age-based grouping, and (3) a biological dentition stage-based grouping. By doing so, we seek to determine which classification method best highlights the strengths and limitations of AI, thereby providing evidence-based guidelines for its application in diverse clinical scenarios.

## 2. Materials and Methods

### 2.1. Study Design

This retrospective, cross-sectional study was conducted using a database of digital lateral cephalometric radiographs acquired between 2018 and 2024 at the University of Medicine and Pharmacy at Ho Chi Minh City, Vietnam. The study protocol was approved by the Institutional Ethical Committee of the University of Medicine and Pharmacy at Ho Chi Minh City, under reference number 1819/DHYD–HDDD. Informed consent was obtained from all patients or their legal guardians, allowing the use of their clinical records for research purposes.

### 2.2. Materials

A final sample of 300 radiographs from Vietnamese orthodontic patients was selected based on the following inclusion criteria: (1) high-resolution images (300 dpi, dimensions 1360 × 1018 pixels) with clear visibility of all necessary anatomical landmarks and (2) the absence of motion artifacts, severe craniofacial deformities, or significant asymmetry. All cephalograms were acquired using a Vatech imaging system (Vatech Inc., Gyeonggi-do, Republic of Korea) and following a standardized acquisition protocol. The sample size was determined a priori to ensure adequate statistical power using established formulas [17], based on an expected average Intraclass Correlation Coefficient (*ICC*) of 0.83, a type I error rate of 5%, and a desired margin of error of 0.05 [18].

### 2.3. Methods

**Figure 1:**
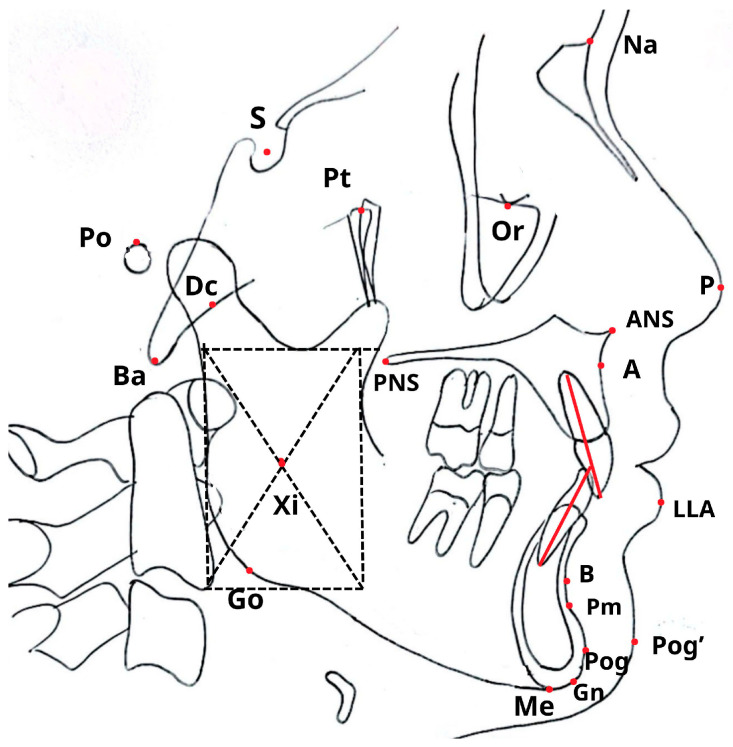
Cephalometric landmarks (see Appendix A.1 for detail explanation).

All 300 cephalograms were evaluated using two approaches: manual tracing by an experienced orthodontist and a fully automated analysis via WebCeph. The spatial locations of the key cephalometric landmarks are shown in Figure 1, with definitions provided in Table A1.

**Figure 2:**
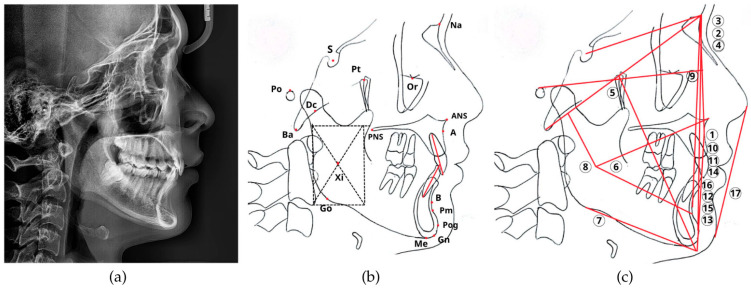
The manual cephalometric analysis workflow. The traditional manual analysis process. (**a**) The original digital radiograph was printed. (**b**) Anatomical structures and landmarks were traced onto an acetate sheet. (**c**) A total of 17 angular and linear parameters (numbered 1–17) indicated by numbered labels: (1) Convexity; (2) ANB; (3) SNA; (4) SNB; (5) Facial axis; (6) Lower facial height; (7) Mandibular plane angle; (8) Mandibular arc; (9) Facial depth; (10) U1–NA (linear); (11) U1–NA (angle); (12) L1–NB (linear); (13) L1–NB (angle); (14) Interincisal angle; (15) L1–APog (linear); (16) L1–APog (angle); (17) Lower lip–E line, were then measured from the tracing using a specialized ruler and protractor. The dash frame was used to determine the geometric center of the mandibular ramus, while the red lines indicate the reference lines for cephalometric analysis.

The manual tracing workflow, illustrated in Figure 2, was conducted by a single calibrated orthodontist with over 10 years of clinical experience. Each digital radiograph was printed at a 1:1 scale on A4 paper to facilitate tracing. Anatomical landmarks were identified and traced onto 0.003-inch acetate paper sheets using a 0.4 mm fine-tip pen over a radiographic viewing box. A specialized cephalometric protractor and ruler were then used to measure 17 parameters (12 angular and 5 linear), as detailed in Table A2. The resulting measurements were digitized and recorded as the manual-tracing reference standard for this study.

**Figure 3:**
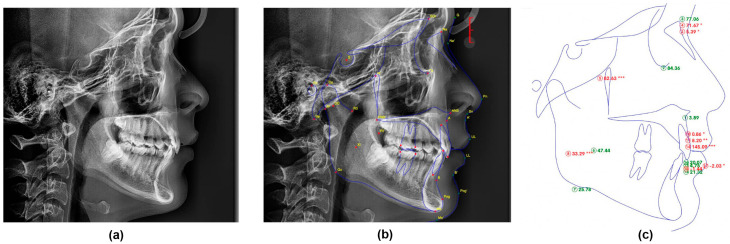
The automated AI-Powered cephalometric analysis workflow. The fully automated analysis process using the WebCeph software. (**a**) The original digital radiograph was uploaded to the system. (**b**) The AI algorithm performed automated landmark identification. (**c**) The software generated the final cephalometric analysis with all 17 parameters automatically calculated. The asterisk indicates the severity of deviation from the normal range: * mild, ** moderate, *** severe.

For the automated method (Figure 3), each original digital radiograph in JPEG format was uploaded to the AI-powered WebCeph software (Version 2.0.0, 2024, Assemble Circle, Gyeonggi-do, Republic of Korea). The software automatically identified cephalometric landmarks and calculated the same 17 parameters as the manual method. To assess the raw performance of the algorithm, all measurements were recorded directly without any manual correction.

### 2.4. Accuracy Assessment and Stratification Strategies

To establish the consistency of the measurement process, intra-observer reliability was assessed using a random subset of 30 radiographs (10% of the total sample) re-analyzed by the same orthodontist after a two-week interval [19]. Reliability was quantified through ICC and Dahlberg’s measurement error ($S_{e}=\sqrt{\sum d^{2}/2n}$) to validate manual tracing as a stable reference standard. AI accuracy was subsequently evaluated using three stratification strategies to identify the most clinically relevant grouping method. These strategies included an overall analysis of the entire cohort, a demographic stratification by chronological age (<18, 18–25, and >25 years) [20], and a biological stratification based on dentition stage, classified as primary-early mixed (<9 years), late mixed (9–12 years), and permanent dentition (>12 years).

### 2.5. Statistical Analysis

All statistical processing was performed using Python (version 3.9) with the Pingouin statistical package (version 0.5.3), and the level of significance was set at p<0.05. The normality of data distribution was first evaluated using the Shapiro–Wilk test to determine the appropriate statistical approach for subsequent comparisons. To ensure statistical independence, the unit of analysis was the individual radiograph (*n* = 300), rather than individual landmarks or measurements. Agreement between manual tracing and WebCeph was quantified using ICC based on a two-way mixed-effects model for absolute agreement, reported with 95% confidence intervals (*CI*). Additionally, Bland–Altman analysis was utilized to visualize the systematic bias and limits of agreement between the two methods.

To compare the primary outcome—absolute measurement difference (∣*Δ*∣)—across subgroups, the Kruskal–Wallis H test was employed, followed by Dunn’s post hoc test with Bonferroni correction, as the error data exhibited a non-normal distribution. The magnitude of these differences was quantified using the Kruskal–Wallis effect size ($\eta_{H}^{2}$), calculated as $\eta_{H}^{2}=(H-k+1)/(n-k)$, where H is the test statistic, k is the number of groups, and n is the total sample size. Effect sizes were interpreted as small ($0.01\leq \eta^{2}<0.06$), medium ($0.06\leq \eta^{2}<0.14$), or large ($\eta^{2}\geq 0.14$) [4]. Finally, interaction plots were generated to qualitatively assess the “Method × Group” interaction and identify specific developmental stages where AI performance significantly deviated from the manual reference standard.

## 3. Results

### 3.1. Sample Characteristics

A total of 300 patients were included in the final analysis. The demographic characteristics and distribution of the sample according to the three stratification strategies are summarized in Table 1; all quantitative values are presented as mean ± standard deviation (*SD*). The sample included 82 males and 218 females.

### 3.2. Intra-Observer Reliability

The assessment of intra-observer reliability, conducted on 30 randomly selected replicates, confirmed excellent consistency for the manual tracing method, validating it as a robust reference standard. The *ICCs* for manual measurements indicated excellent agreement, ranging from *0.942* (L1 to APog linear) to *0.998* (Interincisal angle), with an overall mean *ICC* of *0.981*. Similarly, the random measurement error was clinically insignificant, with Dahlberg’s values ($S_{e}$) ranging from *0.46* to *0.74* units (mm or degrees) for all manual parameters.

Consistent with its computational nature, the automated WebCeph analysis demonstrated slightly higher reproducibility with a mean *ICC* of *0.989* and lower Dahlberg errors (range: *0.35*–*0.56* units), reflecting the inherent algorithmic stability of the software. The detailed reliability statistics for each parameter are presented in Table 2.

### 3.3. Strategy 1—Overall Agreement

Table 3 summarizes the descriptive statistics and reliability analysis for the entire study cohort (*n* = 300). Overall, WebCeph demonstrated a high level of concordance with the manual reference standard, as evidenced by the majority of parameters yielding *ICC* values exceeding 0.80. Within the skeletal category, SNB and Facial Axis exhibited the highest reliability (*ICC* = 0.94 and 0.93, respectively), indicating excellent stability in the assessment of sagittal and vertical skeletal patterns. In contrast, the Mandibular Arc showed the lowest agreement among all 17 measured variables (*ICC* = 0.69; 95% *CI:* 0.61–0.76), reflecting only a moderate correlation. Furthermore, a notable systematic discrepancy was observed for Convexity, where the AI system tended to overestimate the measurement by approximately 1.0 mm compared to the manual method.

Regarding dental measurements, reliability was generally classified as good to excellent, with the L1–APog linear distance demonstrating high agreement (*ICC* = 0.90). However, the L1–APog angle yielded comparatively lower reliability (*ICC* = 0.77), suggesting that the AI algorithm may encounter challenges in precisely identifying the long axis of the lower incisor. Performance in soft tissue analysis remained highly proficient, particularly for the Lower lip to E-plane measurement, which achieved excellent reliability (*ICC* = 0.94; 95% *CI*: 0.92–0.96). This indicates the software’s effectiveness in detecting high-contrast soft tissue profiles, ensuring stable results for external facial analysis.

### 3.4. Strategy 2—Comparison by Chronological Age

Table 4 summarizes the performance of WebCeph across three chronological age groups: Group A1 (<18 years), Group A2 (18–25 years), and Group A3 (>25 years). Consistent with the biological maturation of the craniofacial complex, a general upward trend in mean *ICC* was noted, rising from 0.850 in the pediatric/adolescent cohort to 0.889 in the adult group. Within the skeletal category, agreement remained high for stable landmarks such as SNB and Facial Axis (*ICC* > 0.92), whereas the Mandibular Arc proved more difficult for automated detection. While *ICC* values for the Mandibular Arc were numerically lower in younger patients (0.594) than in adults (0.747), the absolute error comparison failed to reach statistical significance (0.771), indicating that chronological age alone is not a primary source of variability for mandibular skeletal landmarks.

Regarding dental parameters, only the L1–APog linear measurement exhibited a statistically significant difference across groups (p=0.037); however, the associated effect size was minimal ($\eta^{2}=0.015$), suggesting that the practical impact of age on the AI’s precision is clinically limited. Other variables, such as U1–NA angle (p=0.054) and L1–NB angle (p=0.087), approached but did not exceed the significance threshold. In summary, the predominance of non-significant results (16 out of 17 parameters with p>0.05) combined with consistently low effect sizes confirms that chronological age is an insensitive predictor of automated landmark detection accuracy. These findings suggest that age-based stratification fails to fully capture the anatomical variability associated with individual biological development.

### 3.5. Strategy 3—Comparison by Dentition Stage

Stratifying the sample by dentition stage provided granular insight into the AI’s performance, revealing specific vulnerabilities in the primary—early mixed dentition phase that were not fully apparent in the chronological age analysis. As presented in Table 5, the primary-early mixed dentition (Group D1: <9 years) exhibited the lowest reliability across critical skeletal and dental parameters. Most notably, the Mandibular Arc measurement in this group dropped to a significantly low level of agreement (*ICC* = 0.349), contrasting sharply with the stable performance observed in the permanent dentition group (*ICC* = 0.734).

Statistical analysis using the Kruskal–Wallis test confirmed that the dentition stage significantly influences the magnitude of measurement errors (∣*Δ*∣). Specifically, significant differences were found for the Mandibular Plane Angle (p = 0.048), L1–NB linear (p=0.035), and L1–APog linear (p=0.012). Analysis of the effect size ($\eta^{2}$) for these significant parameters indicated a small but statistically distinct impact of dental development on AI accuracy. The L1–APog (linear) demonstrated the largest effect size ($\eta^{2}=0.023$), followed by L1–NB linear ($\eta^{2}=0.016$) and Mandibular Plane Angle ($\eta^{2}=0.014$). Although these effect sizes are classified as small according to Cohen’s guidelines, they highlight a consistent pattern: the transitional anatomy of the mixed dentition introduces a systematic “biological noise” that subtly degrades the precision of automated landmark detection, particularly for the lower incisors and mandibular base.

### 3.6. Interaction Effects—Method × Group

Interaction analysis revealed that the dentition stage strategy possessed superior sensitivity in detecting AI performance variations compared to the Chronological Age approach. While age-based stratification identified significant differences in only one parameter (L1–APog linear), dentition-based analysis uncovered significant disparities in three key variables: Mandibular Plane Angle, L1–NB linear, and L1–APog linear. Furthermore, the magnitude of the interaction was consistently higher in the biological model; for the L1–APog linear parameter, the effect size for dentition stratification ($\eta^{2}=0.023$) was approximately 50% larger than that of chronological age ($\eta^{2}=0.015$). These findings confirm that the biological status of the dentition exerts a more profound influence on AI landmark detection accuracy than demographic age alone.

**Figure 4:**
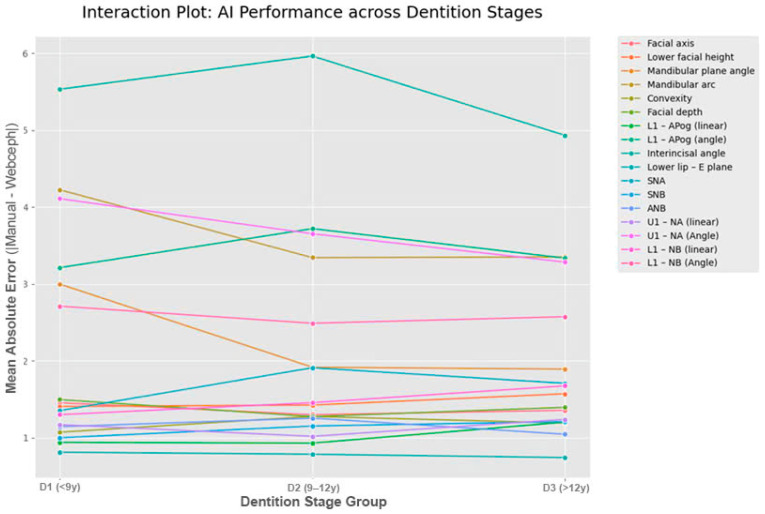
Interaction plot displaying the Mean Absolute Error (∣*Δ*∣) of AI measurement across three dentition stages.

The biological strategy also successfully unmasked critical errors that were otherwise diluted in broader age groupings. For instance, the Mandibular Arc reliability, which appeared moderate in the <18 years age group (*ICC* = 0.594), dropped to a critical level (*ICC* = 0.349) when specifically isolated within the primary-early mixed dentition stage (<9 years). As illustrated in Figure 4, error trajectories for complex parameters—specifically the Mandibular Arc and lower incisor measurements—exhibit a distinct downward slope from the D1 (early mixed) to D3 (permanent) stages, whereas stable skeletal landmarks (e.g., SNA, SNB) remain relatively constant. This interaction pattern reinforces the conclusion that dentition stage is the primary modifier of AI reliability, with measurement stability progressively increasing as the patient transitions to permanent dentition.

## 4. Discussion

### 4.1. Principal Findings

The transition from manual cephalometric tracing to automated, AI-driven analysis represents a significant advancement in orthodontic diagnostics. This study aimed to validate the accuracy of the WebCeph software in a cohort of Vietnamese patients and, crucially, to determine the optimal stratification strategy for evaluating AI performance in growing individuals.

Our findings indicate that WebCeph generally demonstrates a high level of agreement with the manual reference standard for the majority of skeletal and dental parameters (*ICC* > 0.80). This is consistent with recent 2025 studies, which confirmed excellent reliability [20,21,22] (*ICC* > 0.90) for stable skeletal parameters like SNA and SNB, although significant differences in mean values remain between AI and manual methods. However, the central finding of this study is that dentition stage is a more sensitive and biologically relevant predictor of AI accuracy than chronological age. While stratifying by age revealed only minor and mostly non-significant fluctuations in accuracy ($\eta^{2}\approx 0.015$), stratifying by dentition stage unmasked significant performance vulnerabilities ($\eta^{2}\approx 0.023$), particularly in the primary-early mixed dentition phase. Although some differences were statistically significant, the effect sizes were small. This is likely due to the large sample size (*n* = 300), which provides high statistical power to detect even clinically negligible differences.

### 4.2. Reliability Considerations

Before interpreting the AI’s performance, it is essential to establish the validity of the reference standard. In this study, the intra-observer reliability for manual tracing was excellent (mean *ICC* = 0.981), with Dahlberg’s errors consistently below 0.75 mm/degrees. This high level of human consistency ensures that the discrepancies observed between the two methods (*Δ*) are attributable to the limitations of the AI algorithm rather than the variability of the human tracer. This reinforces the credibility of our findings regarding the specific “blind spots” of the AI in the mixed dentition group.

### 4.3. Interpretation of Grouping Strategies Between Age and Dentition

A novel contribution of this study is the direct comparison of two stratification strategies. Previous studies have often relied on chronological age as a convenient proxy for development. However, our results demonstrate that chronological age is a less sensitive predictor for assessing AI reliability, as it fails to account for individual biological variations. When grouped by age (<18 vs. >18), the statistical differences in measurement error were weak and largely insignificant (p>0.05), likely because the “under 18” group is a heterogeneous mix of varying dental stages.

Expanding on this observation, the dentition stage strategy provided a much more granular insight. The interaction analysis revealed that the “primary-early mixed dentition” phase (<9 years) acts as a distinct confounding factor. This was most evident in the Mandibular Arc measurement, where the ICC dropped to a critical level of 0.349 in the primary—early mixed group, compared to 0.594 when grouped simply by age. From a biological perspective, these findings are attributable to the significant ‘anatomical noise’ characteristic of this developmental phase. The coexistence of deciduous roots, developing permanent tooth germs, and active remodeling of the mandibular ramus produces complex overlapping radiodensities that confound automated landmark detection algorithms, particularly in the mandibular region. Furthermore, Zughair et al. recently highlighted that AI-based automatic tracing tends to overestimate certain skeletal values such as SNA and ANB [22,23,24], a phenomenon we observed was more pronounced in the primary-early mixed dentition group due to the difficulty in pinpointing Point A and B during tooth eruption [20,25].

Similarly, the significant effect size found for L1–APog linear ($\eta^{2}=0.023$) in the dentition analysis suggests that the AI struggles to locate the apex of the lower incisors when the root formation is incomplete or obscured by the symphysis maturation—a nuance that was statistically diluted when analyzed by age alone.

Regarding the variability in measurement agreement, certain parameters exhibited relatively wide 95% *CIs*, particularly in the primary-early mixed dentition group. This observation can be attributed to several factors:

Subgroup Sample Size and Statistical Power: While the total sample size (*n* = 300) was robust, the stratification into specific biological stages resulted in smaller subgroups, such as the primary-early mixed dentition group (*n* = 47). Statistically, a smaller sample size within a highly variable population naturally leads to wider *CIs*, reflecting the increased uncertainty when predicting AI performance in this specific developmental window.

Biological Complexity and ‘Fuzzy’ Landmarks: In growing patients, landmarks are not static points but are located in areas of active remodeling [16]. As noted by Zughair et al. in 2025 [22], the accurate localization of Point A and Point B is often compromised by the eruption of permanent incisors and the resorption of primary roots. These ‘fuzzy’ landmarks introduce a high degree of variance in the raw data, which manifests as wider *CIs* in the subsequent reliability analysis. Wide variations in confidence intervals (*CIs*) were observed for parameters such as L1–NB and Interincisal angle, despite the large sample size. This can be attributed to the high biological variability of incisor inclination in the Vietnamese population (bimaxillary protrusion) and the ‘fuzzy’ nature of identifying the lower incisor apex on 2D radiographs, which leads to higher variance in the dataset.

Nature of Constructed Landmarks: Parameters such as the Mandibular Arc rely on constructed landmarks like the Xi point (the geometric center of the ramus). Raby et al. [23] emphasized that AI algorithms often struggle with landmarks that lack clear high-contrast cortical borders [26,27]. The inherent difficulty in consistently identifying the Xi point—combined with the high anatomical variability of the mandibular ramus in children—creates a ‘compounding error’ effect, leading to lower *ICCs* and broader confidence ranges.

Population-Specific Morphological Variation: The wide *CIs* may also reflect the distinct craniofacial morphology of the Vietnamese population, which may differ from the predominantly Caucasian or East Asian datasets used to train the WebCeph algorithm. This ‘ethnic mismatch’ can lead to inconsistent AI performance across different facial types (e.g., Class II vs. Class III), further spreading the distribution of measurement errors.

### 4.4. Comparison with Previous Studies and Generalizability

Most existing validation studies have been conducted on Caucasian or East Asian datasets with predominantly adult samples. By validating WebCeph on a Vietnamese cohort, this study challenges the algorithm with a dataset that may be under-represented in its training phase. The generally high agreement observed suggests that the algorithm has good generalizability across ethnic groups for adult patients.

However, our findings regarding the Mandibular Arc align with the concerns raised by Serafin et al. [24], who noted that constructed landmarks (like the *Xi* point) are inherently less reliable. Our study adds to this body of knowledge by specifying when this unreliability peaks: specifically during the mixed dentition phase, rather than throughout the entire growth period. Our results echo the findings of Raby et al. (2025) [23], who demonstrated that while AI reduces tracing time by nearly 46%, it still demonstrates low reliability for soft-tissue and complex dental parameters unless manual landmark correction is applied. Similarly, Arslan et al. (2025) [21] found that the Interincisal Angle showed the lowest consistency with AI methods, matching the ‘biological noise’ and anatomical complexity we identified in our primary-early mixed dentition cohort.

### 4.5. Clinical Implications

The findings of this study provide substantial evidence for optimizing orthodontic workflows through the strategic integration of AI. For adult patients and those with permanent dentition, WebCeph serves as a reliable and time-efficient diagnostic adjunct, as its performance in the permanent dentition cohort demonstrated high concordance with expert manual tracing (*ICC* > 0.90). In these cases, the software can be confidently utilized to streamline routine cephalometric analysis, allowing clinicians to focus more on treatment planning and patient communication without compromising diagnostic precision.

Conversely, a more cautious approach is required when applying automated analysis to pediatric patients in the primary and mixed dentition stages (<12 years). The identified “U-shaped” performance curve highlights that AI precision is significantly more vulnerable during the early stages of dental development. Therefore, clinicians should prioritize manual verification of specific landmarks—notably the lower incisor apex and the *Xi* point—before finalized measurements are accepted. Relying exclusively on automated outputs in early developmental phases may lead to inaccuracies in assessing mandibular growth patterns or incisor inclination, potentially compromising the early interceptive orthodontic strategy.

### 4.6. Limitations

The clinical implications of these findings offer critical insights for optimizing orthodontic workflows. However, this study has limitations. First, although the sample size was robust, the subgroups for the primary-early mixed dentition were smaller than the adult groups. Second, the AI performance dropped significantly in the mixed dentition group. While this highlights a specific weakness of the software, future studies should focus specifically on training models with larger mixed-dentition datasets to overcome this. Finally, the study was retrospective, and the manual tracing was performed by a single calibrated observer. While this eliminates intra-observer variation, it limits the assessment of how the AI compares to the broader range of variability found among different human clinicians.

## 5. Conclusions

This study demonstrates that while WebCeph exhibits high reliability compared with manual tracing for the majority of cephalometric parameters, its performance is significantly modulated by the biological stage of dentition. Our findings establish that the dentition stage is a more sensitive and biologically relevant predictor of AI accuracy than chronological age.

The stratification strategy revealed that the primary-early mixed dentition phase (<9 years) presents unique anatomical challenges—such as the superimposition of permanent tooth germs and dynamic alveolar remodeling—that significantly degrade AI precision. These critical vulnerabilities, specifically concerning the Mandibular Arc and lower incisor positioning, were largely obscured when performance was evaluated through broad chronological age categories. This underscores the necessity of using dentition stage as a more sensitive and biologically relevant predictor of AI diagnostic precision.

In light of recent 2025 evidence, our results confirm that while AI-driven analysis is a robust and time-efficient tool for adult patients with permanent dentition, it has not yet reached the level of autonomy required to supplant human expertise in growing children. Clinically, manual verification of mandibular and incisor landmarks remains mandatory for patients in the mixed dentition stage to ensure diagnostic precision and avoid errors in growth assessment.

## Figures and Tables

**Table 1:** Demographic characteristics of the study sample (*n* = 300).

Stratification Strategy	Group	*n* (%)	Mean Age ± *SD*
Strategy 1: Overall	Total Sample	300 (100.0%)	18.6 ± 9.7
Strategy 2: Chronological Age	Group A1 (<18 y)	145 (48.3%)	10.2 ± 2.9
	Group A2 (18–25 y)	79 (26.3%)	21.7 ± 2.2
	Group A3 (>25 y)	76 (25.3%)	31.5 ± 5.9
Strategy 3: Dentition Stage	Group D1 (<9 y)	47 (15.7%)	7.1 ± 0.9
	Group D2 (9–12 y)	72 (24.0%)	10.5 ± 1.2
	Group D3 (>12 y)	181 (60.3%)	24.9 ± 7.4

**Table 2:** Detailed *ICC* results for 30 re-measured radiographs with manual and WebCeph.

Parameters	Index	Manual		WebCeph	
		*ICC* (95% *CI*)	Dahlberg ($S_{e}$)	*ICC* (95% *CI*)	Dahlberg ($S_{e}$)
Skeletal	Convexity	0.960 (0.92–0.98)	0.707	0.990 (0.98–1.00)	0.428
	ANB	0.975 (0.95–0.99)	0.552	0.978 (0.95–0.99)	0.517
	SNA	0.987 (0.97–0.99)	0.461	0.989 (0.98–0.99)	0.451
	SNB	0.986 (0.97–0.99)	0.562	0.993 (0.98–1.00)	0.417
	Facial axis	0.988 (0.98–0.99)	0.633	0.994 (0.99–1.00)	0.459
	Lower facial height	0.987 (0.97–0.99)	0.673	0.990 (0.98–1.00)	0.557
	Mandibular plane angle	0.996 (0.99–1.00)	0.559	0.996 (0.99–1.00)	0.461
	Mandibular arc	0.994 (0.99–1.00)	0.469	0.995 (0.99–1.00)	0.430
	Facial depth	0.984 (0.97–0.99)	0.569	0.988 (0.97–0.99)	0.499
Dental	U1–NA (linear)	0.953 (0.90–0.98)	0.629	0.968 (0.93–0.98)	0.509
	U1–NA (Angle)	0.997 (0.99–1.00)	0.471	0.997 (0.99–1.00)	0.389
	L1–NB (linear)	0.961 (0.92–0.98)	0.537	0.989 (0.98–0.99)	0.353
	L1–NB (Angle)	0.995 (0.99–1.00)	0.527	0.995 (0.99–1.00)	0.463
	Interincisal angle	0.998 (1.00–1.00)	0.610	0.998 (1.00–1.00)	0.480
	L1–APog (linear)	0.942 (0.88–0.97)	0.675	0.978 (0.95–0.99)	0.493
	L1–APog (angle)	0.992 (0.98–1.00)	0.618	0.993 (0.98–1.00)	0.452
Soft tissue	Lower lip–E plane	0.970 (0.94–0.99)	0.510	0.978 (0.95–0.99)	0.490

**Table 3:** Comparison of the reliability of cephalometric measurements between WebCeph and manual tracing.

Parameters	Index	Manual (*n* = 300) Mean ± *SD*	WebCeph (*n* = 300) Mean ± *SD*	*ICC*	*CI95*
Skeletal	Convexity	2.5 ± 3.4	3.5 ± 3.7	0.92	0.66–0.97
	ANB	2.6 ± 3.3	3.4 ± 3.2	0.91	0.78–0.96
	SNA	82.7 ± 3.7	83.9 ± 3.5	0.84	0.60–0.92
	SNB	80.0 ± 4.2	80.5 ± 4.1	*0.94*	0.91–0.95
	Facial axis	87.0 ± 4.6	87.1 ± 4.5	*0.93*	0.91–0.94
	Lower facial height	46.3 ± 4.8	46.8 ± 4.5	*0.91*	0.88–0.93
	Mandibular plane angle	26.1 ± 6.3	25.4 ± 6.0	0.89	0.86–0.92
	Mandibular arc	33.7 ± 6.0	34.9 ± 5.1	*0.69*	*0.61*–*0.76*
	Facial depth	87.9 ± 3.7	87.8 ± 3.6	0.87	0.84–0.90
Dental	U1–NA (linear)	5.5 ± 2.8	5.8 ± 3.1	0.87	0.84–0.90
	U1–NA (Angle)	26.7 ± 8.6	24.5 ± 7.7	0.85	0.72–0.91
	L1–NB (linear)	6.2 ± 2.7	7.7 ± 3.3	0.83	0.09–0.94
	L1–NB (Angle)	29.7 ± 7.5	28.4 ± 7.1	0.88	0.82–0.91
	Interincisal angle	119.1 ± 12.6	123.5 ± 11.4	0.87	0.43–0.95
	L1–APog (linear)	4.6 ± 2.8	5.2 ± 3.2	*0.90*	0.82–0.94
	L1–APog (angle)	27.9 ± 5.9	25.3 ± 5.7	*0.77*	0.36–0.89
Soft tissue	Lower lip–E plane	2.8 ± 2.8	2.5 ± 3.1	*0.94*	*0.92*–*0.96*

**Table 4:** ICC values across three chronological age groups.

Parameters	Index	Group A1 (<18)	Group A2 (18–25)	Group A3 (>25)	*p*-Value *
Skeletal	Convexity	0.907	0.928	0.928	0.815
	ANB	0.889	0.931	0.934	0.082
	SNA	0.833	0.837	0.857	0.712
	SNB	0.938	0.937	0.933	0.853
	Facial axis	0.925	0.934	0.926	0.840
	Lower facial height	0.894	0.901	0.926	0.762
	Mandibular plane angle	0.846	0.916	0.933	0.762
	Mandibular arc	0.594	0.727	0.747	0.771
	Facial depth	0.845	0.851	0.922	0.697
Dental	U1–NA (linear)	0.892	0.857	0.851	0.859
	U1–NA (Angle)	0.825	0.895	0.866	0.054
	L1–NB (linear)	0.783	0.838	0.853	0.537
	L1–NB (Angle)	0.842	0.919	0.872	0.087
	Interincisal angle	0.851	0.878	0.888	0.331
	L1–APog (linear)	0.887	0.884	0.917	0.037
	L1–APog (angle)	0.760	0.739	0.769	0.985
Soft tissue	Lower lip–E plane	0.939	0.934	0.951	0.296
Mean ICC		0.850	0.878	0.889	

* *p*-value from Kruskal–Wallis test comparing the absolute error (|Manual-Webceph|) among the three groups.

**Table 5:** *ICC* values across three dentition stage groups.

Parameters	Index	Group D1 (<9 y, Primary-Early Mixed)	Group D2 (9–12 y, Late Mixed)	Group D3 (>12 y, Permanent)	*p*-Value
Skeletal	Convexity	0.866	0.903	0.931	0.137
	ANB	0.875	0.885	0.930	0.117
	SNA	0.787	0.848	0.840	0.064
	SNB	0.925	0.944	0.933	0.682
	Facial axis	0.884	0.941	0.929	0.282
	Lower facial height	0.896	0.904	0.910	0.847
	Mandibular plane angle	0.717	0.893	0.925	0.048
	Mandibular arc	0.349	0.645	0.734	0.378
	Facial depth	0.756	0.890	0.879	0.864
Dental	U1–NA (linear)	0.830	0.874	0.864	0.478
	U1–NA (Angle)	0.824	0.775	0.875	0.143
	L1–NB (linear)	0.727	0.817	0.831	0.035
	L1–NB (Angle)	0.642	0.890	0.899	0.280
	Interincisal angle	0.843	0.817	0.885	0.190
	L1–APog (linear)	0.890	0.875	0.900	0.012
	L1–APog (angle)	0.755	0.715	0.763	0.409
Soft tissue	Lower lip–E plane	0.916	0.937	0.945	0.454
Mean ICC		0.791	0.859	0.882	

## Data Availability

The original contributions presented in this study are included in the article. Further inquiries can be directed to the corresponding author.

## Appendix A

### Appendix A.1

This appendix provides a comprehensive list and detailed definitions of the 20 cephalometric landmarks utilized in this study. To ensure the reproducibility of both the manual reference standard and the AI-based automated tracing, each landmark—comprising skeletal, dental, and soft tissue points—was identified based on the standardized anatomical descriptions presented in Table A1. These definitions served as the foundational criteria for the calibrated orthodontist and the WebCeph algorithm during the comparative analysis.

**Table A1:** Definitions of cephalometric landmarks.

No.	Landmark	Definition
1	Sella (S)	The midpoint of the sella turcica, representing the center of the pituitary fossa.
2	Nasion (Na)	The junction of the frontal and nasal bones at the nasofrontal suture.
3	Pterygoid point (Pt)	The most posterior point on the contour of the pterygomaxillary fissure (the “teardrop” shape).
4	Basion (Ba)	The most inferior and anterior point on the anterior margin of the foramen magnum.
5	Dorsum condyle (Dc)	The most posterior-superior point on the mandibular condylar head.
6	Porion (Po)	The uppermost point on the external auditory meatus; used to define the Frankfort Horizontal plane.
7	Orbitale (Or)	The lowest point on the infraorbital rim; paired with Po to form the Frankfort Horizontal plane.
8	Xi point (Xi)	The geometric center of the mandibular ramus, determined by bisecting its height and width.
9	Gonion (Go)	The most posterior-inferior and outward point at the angle of the mandible.
10	Point A (A)	The deepest point on the curvature between the anterior nasal spine and the maxillary alveolar crest.
11	Point B (B)	The deepest point on the concavity between the mandibular alveolar crest and pogonion.
12	Supramentale (Pm)	The point where the curvature from the lower incisor alveolar crest transitions to the contour of the bony chin, slightly below Point B.
13	Pogonion (Pog)	The most anterior point on the bony chin.
14	Gnathion (Gn)	The midpoint between Pogonion and Menton on the antero-inferior contour of the mandible.
15	Menton (Me)	The most inferior point on the bony chin.
16	Anterior Nasal Spine (ANS)	The most anterior point of the nasal spine of the maxilla.
17	Posterior Nasal Spine (PNS)	The most posterior point of the hard palate, located at the end of the palatine bone.
18	Pronasale (P)	The most anterior point of the nasal tip.
19	Lower Lip Anterior (LLA)	The most anterior point on the convexity of the lower lip.
20	Soft tissue Pogonion (Pog’)	The most anterior point on the soft tissue chin.

### Appendix A.2

Following the landmark identification, Table A2 provides the detailed definitions and categorized descriptions of the 17 cephalometric parameters (skeletal, dental, and soft tissue) analyzed in this research. Each parameter is defined by specific anatomical indices and reported in standardized units—millimeters (mm) for linear distances and degrees (°) for angular measurements. These definitions ensure a precise and objective framework for comparing the measurements derived from the WebCeph AI system against the manual reference standard.

**Table A2:** Definitions and categorization of cephalometric parameters.

Parameters	Index	Description	Unit
Skeletal	Convexity	Distance between point A and the facial plane	mm
	ANB	Angle determined by points A, N, and B	(°)
	SNA	Angle determined by points S, N, and A	(°)
	SNB	Angle determined by points S, N, and B	(°)
	Facial axis	Angle between the facial axis and Basion–Nasion	(°)
	Lower facial height	Angle from anterior nasal spine (ANS) to the center of the ramus (Xi) to Pm	(°)
	Mandibular plane angle	Angle between the mandibular plane (Sgo–Me) to Frankfort horizontal	(°)
	Mandibular arc	Angle between the corpus axis (Xi–Pm) and condyle axis (Xi–Dc)	(°)
	Facial depth	Angle between the facial plane (Na–Pog) and Frankfort Plane (Po–Or)	(°)
Dental	U1–NA (linear)	Perpendicular distance from the tip of the maxillary incisor to the plane between points N and A	mm
	U1–NA (angle)	Angle formed by the intersection of the maxillary incisor axis to the plane between points N and A	(°)
	L1–NB (linear)	Perpendicular distance from the tip of the mandibular incisor to the plane between points N and B	mm
	L1–NB (angle)	Angle formed by the intersection of the mandibular incisor axis to the plane between points N and B	(°)
	Interincisal angle	Angle formed by the intersection of the mandibular incisor axis to the maxillary incisor axis	(°)
	L1–APog (linear)	Distance from the tip off the lower incisor to the line defining the jaws, the “A-Pog” plane	mm
	L1–APog (angle)	Angle between the long axis of the lower incisor and the “A-Pog” plane	(°)
Soft tissue	Lower lip–E plane	Distance between the lower lip and the esthetic (nose-chin) plane	mm
